# Reduced interpersonal head synchrony in youth at clinical high risk for psychosis

**DOI:** 10.1017/S0033291725102754

**Published:** 2025-12-29

**Authors:** Juliette Lozano-Goupil, Victor Pokorny, Jason Schiffman, Steven M. Silverstein, James M. Gold, James A. Waltz, Lauren M. Ellman, Gregory P. Strauss, Elaine F. Walker, Albert Powers, Philip R. Corlett, Scott W. Woods, Vijay A. Mittal

**Affiliations:** 1 Department of Psychology, Northwestern University, Evanston, IL, USA; 2 Department of Psychological Science, 4201 Social and Behavioral Sciences Gateway, University of California, Irvine, CA, USA; 3 Departments of Psychiatry, Neuroscience and Ophthalmology, University of Rochester Medical Center, Rochester, NY, USA; 4 Maryland Psychiatric Research Center, Department of Psychiatry, University of Maryland School of Medicine, Baltimore, MD, USA; 5 Department of Psychology & Neuroscience, Temple University, Philadelphia, PA, USA; 6 Departments of Psychology and Neuroscience, University of Georgia, Athens, GA, USA; 7 Department of Psychology and Program in Neuroscience, Emory University, Atlanta, GA, USA; 8 Department of Psychiatry, Yale University, New Haven, CT, USA

**Keywords:** automated tools, head movements, interpersonal synchrony, psychosis risk, social functioning, virtual clinical interview

## Abstract

**Background:**

Impaired social functioning is commonly observed in youth at clinical high risk (CHR) for psychosis. Interpersonal synchrony, defined as the temporal alignment of movement between interacting partners, is a key component of successful social interactions. This study aimed to investigate interpersonal head synchrony in naturalistic virtual settings among CHR individuals using automated video analysis tools.

**Methods:**

We analyzed short video recordings from virtual clinical interviews involving 116 participants including 50 CHR participants, 36 individuals with sub-threshold positive symptoms (SUB), and 30 healthy controls (HC). Vertical head movement time series were extracted using an open-access video-based head-tracking tool. Interpersonal head synchrony was computed using Windowed Cross-Correlation to assess group differences and associations with clinical symptoms and functioning.

**Results:**

CHR participants showed significantly reduced strength of synchrony compared to HC (β = −0.05, 95% CI [−0.09, −0.02], *p* = .004), although 14% of variance in strength of synchrony was attributable to assessor identity. No significant group differences were found for delay of synchrony. Within the CHR group, delay of synchrony was positively associated with social anhedonia (*r* = 0.29). Strength of synchrony correlated with better social (*r* = 0.33) and role (*r* = 0.28) functioning.

**Conclusion:**

Our findings suggest that impaired interpersonal head synchrony is already present in the psychosis-risk state and relates to negative symptoms and social and role functioning. These findings support the utility of nonverbal synchrony as a potential biomarker and demonstrate the feasibility of automated tools and virtual assessments to study social processes in at-risk populations.

## Introduction

Interpersonal synchrony refers to temporal alignment of movements in interacting partners (Bernieri & Rosenthal, 1991). This social behavior is a critical process of social interactions with studies demonstrating that synchronous behaviors promoted affiliation (Hove & Risen, 2009), cooperation (Wiltermuth & Heath, 2009), and connectedness within groups (Lumsden, Miles, & Macrae, 2014). Interpersonal synchrony is thought to be disrupted in psychotic psychopathology (Dean, Scott, & Park, 2021; Fattal, McAdams, & Mittal, 2025). For example, joint action paradigms utilizing wrist pendulums have shown less motor synchrony in individuals with schizophrenia compared to controls when explicitly instructed to synchronize their movements with a partner, but similar behavior was observed without explicit instructions, known as the nonintentional synchrony condition (Del-Monte et al., 2013; Varlet et al., 2012). In addition, laboratory-assessed interpersonal synchrony was used as a predictor of classification of 59 participants as either a person with schizophrenia or as healthy controls (HCs) with 93% accuracy and 100% specificity (Słowiński et al., 2017). Analyzing more natural social interactions, individuals with schizophrenia have demonstrated reduced spontaneous interpersonal synchrony during communication, especially in torso and head movements (Kupper, Ramseyer, Hoffmann, & Tschacher, 2015; but see also Lavelle, 2012). Authors also found that less synchrony was associated with severity of negative symptoms and general functioning. Interpersonal synchrony has also been studied in social rehabilitation contexts, showing that body-oriented therapy could significantly improve interpersonal synchrony during clinical interviews, suggesting that this social motor process could be learned (Galbusera, Finn, & Fuchs, 2018). Taken together, these studies suggest that interpersonal synchrony is impaired in schizophrenia spectrum disorders; however, there are limited studies that have shown similar impairments in individuals who are at clinical high risk (CHR) for psychosis.

Interpersonal factors have been shown to be important prospective predictors of risk for psychosis (Doborjeh et al., 2023). Sensory-motor and social processes have been shown to be impaired in individuals at CHR for psychosis (Mittal & Walther, 2019; Schiffman et al., 2004; Walker, Savoie, & Davis, 1994), potentially suggesting impaired spontaneous interpersonal synchrony. To date, the only study of synchrony in CHR was focused on neural synchronization, wherein partners completed a cooperation task and interbrain synchrony was reduced in the right inferior frontal gyrus, assessed using functioning near-infrared spectroscopy hyperscanning technology (Wei et al., 2023). Additionally, a study of individuals with psychotic-like experiences found preserved smiling mimicry, an emotional facial feature of spontaneous interpersonal synchrony (Riehle & Lincoln, 2017). No study to date has assessed spontaneous interpersonal motor synchrony during communication in individuals at CHR for psychosis.

The recent development of technologies and methodologies from diverse scientific fields like Computer Vision and Human Movement Sciences allows for the precise quantification of spontaneous interpersonal behavior in natural interactive contexts. In the field of clinical psychology, behavioral coding has been the standard method of assessing interpersonal synchrony (Bernieri, Reznick, & Rosenthal, 1988; Cappella, 1997; Trevarthen & Daniel, 2005). However, these behavioral coding methods are time-consuming, and the subjectivity and the reliability of ratings are critical limiting factors. To overcome these limitations, automatic methods using video-based tracking techniques have been used to assess face-to-face conversations (Kupper et al., 2015; Ramseyer & Tschacher, 2014). The most common automatic method is Motion Energy Analysis (MEA; Ramseyer, 2020), a method based on evaluating differences in grayscales pixels on videos. While MEA efficiently assesses nonverbal behavior and interpersonal synchrony in clinical interviews, it may not capture more nuanced and complex movements such as head nods. For example, when the head is detected as the region of interest, MEA combines head movements in all directions and also includes additional actions, such as scratching or displacement behaviors. To address this limitation, new machine learning-based tools enable automatic assessment of specific body movements (Lugaresi et al., 2019). These open-access tools require minimal technical expertise and can handle low-resolution video from basic devices like webcams and smartphones. A recent validation study compared the use of MEA and an open-access body-tracking program in the analysis of spontaneous interpersonal synchrony during unstructured conversation, and both approaches produced similar results (Fujiwara & Yokomitsu, 2021). However, authors explicitly advised using a body-tracking program over MEA when researchers wish to focus on synchrony in specific body parts, such as head nods (which are the major body parts visible on zoom videos).

In the present study, we investigated interpersonal head synchrony in a sample of CHR, individuals with sub-threshold positive symptoms (SUB) and young HC participants. Using a range of control groups, including those with elevated vulnerability to psychosis but not quite CHR (i.e., SUB), allowed us to investigate these socio-motor markers at different points on the psychosis spectrum. Each participant was recorded during an online structured clinical interview, and the first 10 minutes of footage were processed using an open-access machine learning-based multiperson head-tracking program. We analyzed the head movement time-series of both participants and assessors to calculate each dyad’s interpersonal head synchrony in the vertical dimension (i.e., head nods). We hypothesized, based on previous findings of reduced interpersonal synchrony in schizophrenia (Dean et al., 2021), that synchrony would be reduced in the CHR group. Furthermore, given prior findings suggesting links between alterations in interpersonal synchrony and clinical symptoms (Kupper et al., 2015), we posited that these deficits would be associated with negative symptoms, as well as social functioning.

## Method

### Participants

A total of 116 participants with usable video data were selected from a larger sample recruited across six study sites (Northwestern University, Yale University, University of Georgia, Temple University, Emory University, and the University of California Irvine). Recruitment materials included printed and electronic fliers, radio and public transportation advertisements, and mail-outs to community health care providers. Participants were also referred from other ongoing CHR studies. Participants were deemed to be at CHR (*N* = 50) based on the Structured Interview for Psychosis-Risk Syndromes (SIPS) criteria (Miller et al., 2003). Participants who did not meet the criteria for a psychosis risk syndrome were categorized into two groups: a sub-threshold group (SUB; *N* = 36) and a HC group (*N* = 30). The SUB group consisted of individuals who reported some positive symptoms on the SIPS but did not meet the criteria for a psychosis risk syndrome, also named help-seeking controls. The sociodemographic characteristics of the sample are presented in Table 1. The study was approved by the Institutional Review Board of Northwestern University. All adult participants provided informed consent. Minors provided written assent, and their parents or guardians provided written consent.

**Table 1.** Sociodemographic characteristics, clinical symptoms, and social and role functioning of participants

Characteristics	CHR (*N* = 50)	SUB (*N* = 36)	HC (*N* = 30)	Statistics
Age	22.9 ± 3.71	22.7 ± 4.20	22.5 ± 3.86	F(2,112) = 0.119, *p* = .888
Sex, % female	62	47.2	60	*X*^2^ (2) = 2.02, *p* = .364
Race, %				*X*^2^ (10) = 20.2, *p* = .027
African American	12	16.7	6.7	
Asian	16	25	33.3	
Caucasian	48	52.8	43.3	
More than one race	20	2.8	6.7	
Not reported	4	2.8	0	
SIPS				
Positive symptom	11.3 ± 3.30	4.67 ± 2.33		
Negative symptoms	8.77 ± 5.93	5.8 ± 4.48		
Disorganized symptoms	4.35 ± 2.61	2.25 ± 1.87		
Global role functioning	7.26 ± 2.04	7.89 ± 1.62	9.03 ± 0.94	
Global social functioning	6.98 ± 1.44	7.64 ± 1.66	8.86 ± 0.95	

*Abbreviations:* CHR, clinical high risk for psychosis participants; SUB, help seeking control participants; HC, healthy control participants; SIPS, structured interview for psychosis-risk syndromes.

### Measures

Clinical interviews and assessments were conducted online by trained staff, advanced doctoral students, and postdoctoral professionals. The SIPS version 5.6.1 was administered by assessors who passed official SIPS training certified by the creator of the scale and was used to detect the presence of a psychosis-risk syndrome and to determine the CHR status. Thirty-two different assessors conducted the clinical interviews (See Supplementary Materials for details about the assessors – Table S1). The Structured *Clinical Interview for DSM-5, Research Version* (SCID; First, 2015) was used to determine the presence of other mental disorders and determine participant inclusion status.

Social and role functioning in the month prior to the study was assessed via the Global Functioning Scale: Social and Role (GFS/R) scales (Cornblatt et al., 2007). The GFS queried peer relationships, peer conflict, and family involvement. The GFR assessed performance and amount of support needed in the individuals’ primary role.

### Video-based tracking of head

The first 10 minutes of online clinical interviews were used to assess head movements and interpersonal synchrony. During these first 10 minutes, the assessor collected demographic information. The interview content was generally neutral and uniform across participants. Thin slices of behavior (1–10 minutes) have been shown to be sufficient for identifying alterations in social behavior, such as facial expressions (Gupta et al., 2022) and head movements in CHR individuals (Lozano-Goupil et al., 2025). Videos displayed both faces side-to-side (the participant and the assessor) and had a frame rate of 25 Hz.

Footage was processed using a reliable multiperson simple pose tracking, called YOLOv8 (Jocher, Chaurasia, & Qiu, 2023) (from Ultralytics). YOLOv8 estimates 17 2D [x, y] body and face landmarks of several people frame-by-frame from a 2D video. We used Python code from the *EnvisionBOX* to run the YOLOv8 program (Pouw, 2024). The nose (landmark n°0) of both individuals on the video was selected for measuring head movements. The pose-tracking program outputted two time series representing the position trajectories of the nose for each individual present on the video along the x and y axes. The x-axis time series represented horizontal movements, and the y-axis time series represented vertical movements. We focused our analysis specifically on the y-axis movements that represent head nods. Vertical head movements were selected over horizontal movements primarily for practical reasons. During the virtual clinical interviews, assessors frequently moved their heads from side to side while reading questions on their screens, reducing the spontaneity of horizontal movements. Vertical movements, in contrast, capture head nods, which are meaningful nonverbal signals of engagement, agreement, and social responsiveness (Aburumman, Gillies, Ward, & Hamilton, 2022; Benoit & Caplier, 2005). We detected outliers in time series by using median outlier method (i.e., value more than three scaled median absolute deviations were removed). Outlying data points were replaced via linear interpolation. We then applied a median filter with an order of 15 frames. To normalize the data between participants, we performed centering (subtracting the mean) and rescaling (dividing by the standard deviation) on each timeseries.

Total amount of head movement per participant was computed by summing the absolute position values at every timepoint across the y-axis.

### Quantification of interpersonal synchrony

**Figure 1.**
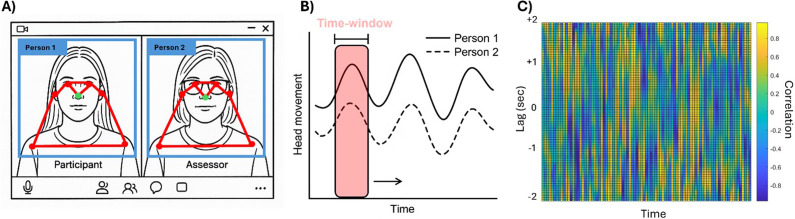
Methodological procedure. (A) Model of the zoom screen used with the YOLOv8 multiperson pose tracking and the nose landmark in green. (B) Model of the nose position timeseries of person 1 and person 2 on the screen and the moving time-window that were used for cross-correlations. (C) Example of the visual output of the Window Cross-Correlation (Lag/Delay versus Time).

The quantification of interpersonal synchrony corresponded to the procedures outlined in Bhatia et al. (2019). We used the Window Cross Correlation (WCC) method to estimate time varying correlations between two time series. In WCC, the time series are split into overlapping segments within a moving time-window (Boker, Rotondo, Xu, & King, 2002). Cross-correlations for time-lags up to 2 s (in both directions) in time-windows of 90 frames (3.6 s) with an overlap of 50% (45 frames) were computed on nose timeseries (y-axis) of both faces (see Figure 1 for an example of the methodological procedure). Cross-correlations were then transformed via Fisher’s Z. It is worth noting that previous studies have used varying parameters for WCC (e.g., window size and lag), which can influence synchrony results. We tested two additional time-windows (5 s and 7 s) that yielded similar results (see Supplementary Materials – Tables S2 and S3).

A peak picking algorithm was then used to analyze the pattern of change in the cross-correlation between both individuals (Boker et al., 2002). The output of the peak picking algorithm is a list of maximum cross-correlations with their corresponding time lag. We then computed the median peak correlation and time lag across the time-windows. While peak correlation indicates the strength of synchrony, the time lag indicates the delay (in seconds) and direction of synchrony (i.e., the leader/follower status). Positive correlation indicates that the direction of the head movement of both the participant and the assessor is the same, whereas negative correlation indicates that the direction of the head movement of both the participant and the assessor is changing out of phase. A positive lag indicates that the assessor’s movement is ahead of the participant’s movement, and a negative lag indicates that the participant’s movement is ahead of the assessor’s movement (See Figure 1C). WCC and peak picking analyses were conducted using Matlab2024b.

### Control with pseudo-synchrony

To assess the significance of measured synchrony, we created 100 surrogate datasets by randomly shuffling the movement segments of the original data, thereby disrupting the temporal alignment between both interactive partners (Kupper et al., 2015; Ramseyer & Tschacher, 2014). This approach preserved the structure of individual behavior while removing the actual interpersonal exchange. Synchrony calculated from these shuffled datasets – termed pseudo-synchrony – was computed using the same method as for the original data.

### Statistical analyses

To test for a group difference in total amount of head movement, we conducted a one-way ANOVA with 3 groups (HC, SUB, and CHR).

To test our first hypothesis on interpersonal synchrony, we first checked for real measured synchrony by comparing the genuine synchrony to the pseudo-synchrony with a dependent t-test. Several clinical interviews had been conducted with the same assessors, leading participants to be nested within assessors. To account for this nesting, we used linear mixed effect models with assessor entered as a random effect and fixed effects of group, participant sex, assessor sex, and participant sex by assessor sex interaction. We include sex in the models because previous work has shown that sex can affect interpersonal synchrony (Vicaria & Dickens, 2016; Zhao et al., 2020). To improve precision regarding sex dynamic on synchrony, we also distinguished between different dyad types: Male–Male, Female–Female, Male (assessor)–Female (participant), and Female (assessor)–Male (participant). Two-way (3 groups × 4 dyad types) ANOVAs were then conducted.

To test our second hypothesis regarding the association between symptoms and interpersonal synchrony, we performed Pearson correlation analyses within CHR and within pooled CHR–SUB. We also tested for associations between interpersonal synchrony and global social and role functioning in the full sample. Correction for multiple comparisons was applied using the false discovery rate (FDR) method (Benjamini & Hochberg, 1995). All statistical tests were performed in RStudio 2024.12.1.

## Results

Groups did not significantly differ in the amount of head movement produced during the interview clip (F(2, 113) = 0.915, *p* = .404). Thus, any reductions in synchrony are unlikely to be attributable to generally reduced head movements in CHR.

The strength of synchrony we observed was significantly higher (1.16 ± 0.08) than the pseudo-synchrony (1.13 ± 0.11) (t(115) = 3.41, *p* < .001). Thus, the synchrony measured during the zoom clinical existed above a level of chance and stems from the sensorimotor exchange happening spontaneously during an interaction.

CHR exhibited lower strength of synchrony than controls (see Table 2). Post hoc pairwise comparisons (FDR-corrected) showed no significant differences between SUB and HC (*t* = 1.19, *p* = .24) or between SUB and CHR (*t* = −1.64, *p* = .16). The effects of participant sex, assessor sex, and the interaction between participant sex and assessor sex were not significant. The intraclass correlation coefficient (ICC), a measure of linear dependence within the group, showed that 14.1% of the total variation in strength of synchrony was attributable to the assessor (see Supplementary Materials – Figure S1).

**Table 2.** Linear mixed models for the strength and delay of synchrony estimate

Strength of synchrony	Fixed effects			Random effects		
	Estimates	95% CI	*p*	Variance	SD	ICC
Intercept	1.18	[1.15–1.22]	**< .0001**			
Group [SUB]	−0.02	[−0.07–0.02]	.230			
Group [CHR]	−0.05	[−0.09 – −0.02]	**.004**			
Participant sex [M]	−0.01	[−0.05–0.03]	.657			
Assessor sex [M]	0.02	[−0.03–0.07]	.396			
Participant sex [M] × assessor sex [M]	−0.02	[−0.08–0.04]	.501			
Assessor				0.0009	0.032	0.141
Residual				0.0059	0.077	
**Delay of synchrony**						
Intercept	−0.01	[−0.11–0.14]	.817			
Group [SUB]	−0.00	[−0.17–0.11]	.636			
Group [CHR]	0.06	[−0.07–0.19]	.393			
Participant sex [M]	−0.09	[−0.23–0.05]	.208			
Assessor sex [M]	−0.06	[−0.20–0.08]	.415			
Participant sex [M] × assessor sex [M]	0.02	[−0.20–0.23]	.878			
Assessor				0.000	0.000	0.00
Residual				0.080	0.283	

*Abbreviations:* CHR, Clinical High Risk for psychosis participants; SUB, help seeking control participants. P-values in bold are statistically significant (*p* < .05).

For delay of synchrony, the effects of group, participant sex, assessor sex, and the interaction between participant sex and assessor sex were not significant (see Table 2). The ICC of the assessor was close to null.

Concerning the sex dynamic, the ANOVA on strength of synchrony revealed no significant main effect of dyad type (F(104) = 0.85, *p* = .47, ges = 0.024) and no significant interaction between group and dyad type (F(104) = 0.58, *p* = .75, ges = 0.032), while the main effect of group remained significant (F(104) = 4.39, *p* = .02, ges = 0.078). Similarly, the ANOVA on delay of synchrony showed no significant main effect of group (F(104) = 1.00, *p* = .37, ges = 0.019), dyad type (F(104) = 1.15, *p* = .33, ges = 0.032), or interaction (F(104) = 0.96, *p* = .46, ges = 0.052). Post hoc tests revealed no significant differences between dyad types for either measure, indicating that the gender composition of the dyads did not significantly influence interpersonal synchrony in this sample.

**Figure 2.**
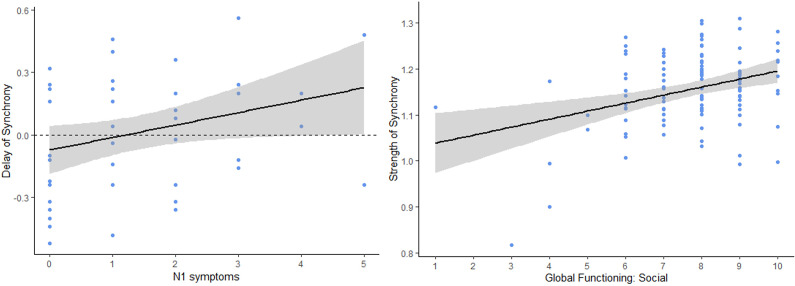
Left: Significant correlation scatter plot between delay of interpersonal synchrony (in seconds) and N1 symptoms (i.e., social anhedonia) in the CHR group. Positive delay of synchrony corresponds to the participants following assessors’ movements and negative delay of synchrony corresponds to the participants leading assessors’ movements. Right: Significant correlation scatter plot between strength of synchrony and the Global Functioning Social scale in the full sample.

Concerning our second hypothesis of interpersonal synchrony being linked to symptoms among the CHR participants, social anhedonia (SIPS N1) was significantly correlated with delay of synchrony (*r* = 0.29, *p* = .044, *p.*_adjust_ = .78) (See Figure 2). This correlation indicated that higher social anhedonia was related to greater positive synchrony delay. In other words, individuals at CHR with greater social anhedonia followed the assessors’ movements with a longer lag. However, this correlation did not survive FDR correction. Additionally, this correlation did not persist when SUB participants were included (*r* = 0.09, *p* = .44, *p.*_adjust_ = .91).

Strength of synchrony was also significantly correlated with the Global Functioning Social (GFS) scale (*r* = 0.33, *p* < .001, *p.*_adjust_ < .01) and the Global Functioning Role (GFR) scale (*r* = 0.28, *p* < .01, *p.*_adjust_ < .01) in the full sample, showing that participants with a higher level of social and role functioning exhibited a stronger synchrony with their assessor during their interaction. However, no significant correlations were found with the delay of synchrony (GFS: *r* = −0.11, *p* = .22, *p.*_adjust_ = .22; GFR (*r* = −0.15, *p* = .10, *p.*_adjust_ = .13).

## Discussion

We examined interpersonal head synchrony during online clinical interviews of individuals at CHR for psychosis, individuals with sub-threshold positive symptoms, and HCs. Our primary finding was that CHR for psychosis were significantly less strongly synchronized with their assessors than the HCs. The model also showed that strength of synchrony was not driven by sex of participant, either sex of the assessor or the interaction between both. Although there is no study to date on interpersonal synchrony in CHR for psychosis to compare these findings, several underlying mechanisms could explain this alteration (Dean et al., 2021). Interpersonal synchrony requires basic sensory, perceptual, and attentional abilities during the input stage that might be altered in CHR (Brewer et al., 2006; Kafadar et al., 2020). In addition, the output stage of producing a behavior could also be impaired. Motor deficits are well described in the prodrome and can manifest as unusual motor activity (e.g., spontaneous dyskinesias), psychomotor slowing, catatonia, and abnormal sway, but also as more impaired social behavior such as lack of hand gestures and head movements during conversation (Dean et al., 2021; Lozano-Goupil & Mittal, 2025; Mittal & Walther, 2019). However, in the present study, we did not observe reduced head movements in CHR. These results agree with a previous study finding of similar total amount of head movements in individuals at CHR for psychosis (Lozano-Goupil et al., 2025). Beyond these perceptual and motor components, another potential social explanation of these results concerns the similarity between partners (Rabinowitch & Knafo-Noam, 2015; Valdesolo, Ouyang, & DeSteno, 2010). According to the dynamic systems theory (Schmidt, Fitzpatrick, Caron, & Mergeche, 2011), dyads with more similar behavioral dynamics should achieve synchrony more quickly and stably. It is therefore possible that HC participants, who are more similar to assessors than CHR participants in terms of absence of psychosis risk, may synchronize more easily, although it has not yet been empirically demonstrated. It is also important to note that the present task involved an assessor–participant interaction, a socially asymmetrical context that likely differs from peer-to-peer interactions. Such role differences may influence how synchrony unfolds, as participants may adopt more passive behavior when interacting with an assessor. On the other hand, social cognition may also be a relevant component, as interpersonal synchrony can reflect shared understanding of the emotional and intentional states of one’s partner. A breakdown in social cognition, such as failing to detect subtle changes in the partner’s behavioral cues, could disrupt synchrony. For example, Gupta et al. (2022) found that CHR spent less time fixating on gestures compared to controls. Thus, it is possible that CHR spent less time focusing on the assessor’s head movements during this interaction, reducing the strength of synchrony. Finally, post hoc analyses showed that SUB participants did not differ from either group. Including SUB participants increases comparability on general psychopathology while preserving CHR specificity (Millman, Gold, Mittal, & Schiffman, 2019). These results suggest that impaired synchrony is specific to CHR individuals rather than a general effect of subthreshold positive symptoms or non-CHR psychopathology.

We did not observe significant differences in synchrony lag between groups. Lavelle (2012) found similar results comparing in-person social interactions between pairs of individuals with schizophrenia with an experimenter or HCs with an experimenter. While disrupted timing and time perception has been extensively investigated in schizophrenia (Carroll, O’Donnell, Shekhar, & Hetrick, 2009; Densen, 1977), studies have demonstrated that those processes could alter intentional interpersonal synchrony but to a lesser extent the nonintentional (i.e., spontaneous) interpersonal synchrony (Słowiński et al., 2017; Varlet et al., 2012). Moreover, evidences for timing deficits in the prodrome are still scare (Osborne et al., 2021).

Social interactions are inherently bidirectional and both interactors influence one another (Schmidt et al., 2011). We observed meaningful variability between assessors in terms of strength of synchrony between dyads. Indeed, 14,1% of the total variance of strength of synchrony was attributable to the assessors. Behavioral studies on dyadic interactions involving multiple interactive partners rarely consider these external individual characteristics that can influence the findings (Vicaria & Dickens, 2016). Indeed, intrapersonal factors can influence interpersonal synchrony such as personality (Arellano-Véliz, Jeronimus, Kunnen, & Cox, 2024) and motivation (Lumsden et al., 2012). This assessor variability in synchrony is important to consider because it may influence clinician-ratings and affect assessor–assessee rapport.

We found that delay of synchrony was significantly associated with the negative symptom ‘social anhedonia’ in the CHR group, meaning that participants with higher social anhedonia symptoms followed their assessor’s head movements. The higher the negative symptoms, the longer the lag. Thus, it would appear that interactive partners could detect abnormalities on a nonverbal level. Although negative symptoms are also present and variable in SUB participants, the significant correlation between delay of synchrony and social anhedonia (SIPS N1) in CHR individuals was no longer observed in the pooled CHR–SUB group. This suggests that the association is specific to CHR participants, for whom clinically meaningful negative symptoms impact interpersonal synchrony. Including SUB participants, who have lower average N1 scores, may have diluted the overall correlation. Moreover, we found an additional significant correlation between strength of synchrony and social and role functioning in the present study. Kupper et al. (2015) similarly found that head synchrony of individuals with schizophrenia was related to social impairment and especially to nonverbal social skills and eye contact. Hence, lack of synchrony may be a crucial factor influencing performance in social behavior (Gallese & Sinigaglia, 2011; Varlet et al., 2012), making it a prime target for future interventions. For example, it has been experimentally demonstrated that subliminal social priming could improve interpersonal synchrony in schizophrenia and later lead to a better relationship quality with an interactive partner (Raffard et al., 2015). Finally, it is important to note that synchrony is not always beneficial: excessive synchrony may reduce self-regulation of affect (Galbusera, Finn, Tschacher, & Kyselo, 2019), potentially intensifying the self–other distinction difficulties observed in schizophrenia (van der Weiden, Prikken, & van Haren, 2015). Therefore, both reduced and overly increased synchrony may reflect maladaptive interpersonal processes.

There are several limitations of the present study to consider. First, we relied exclusively on virtual clinical interviews. Although it has been recently demonstrated that motor synchrony could also arise spontaneously during a virtual social interaction between two strangers and may share similar social effects as face-to-face interactions (Gvirts, Ehrenfeld, Sharma, & Mizrahi, 2023), an important next step will be to compare our results with in-person interactions. Furthermore, high video resolution has been shown to improve interpersonal synchrony (Diao, Arboleda, & Raake, 2024a; Diao, Arevalo Arboleda, & Raake, 2024b). We were unable to measure or manipulate this factor in the present study, which may have influenced our synchrony measurement. However, although analyses using different time-window parameters yielded similar results, parameter selection may still affect specific outcomes. To provide some guidance, prior work suggested that smaller windows, combined with nontransformed data and light smoothing, often produce higher synchrony values and stronger clinical outcomes (Behrens, Moulder, Boker, & Kret, 2020; Schoenherr et al., 2019). Finally, as we lack detailed speech annotations, we could not investigate how speech content might explain the difference in interpersonal head synchrony between groups. Future research should incorporate speech–event annotation to better contextualized synchrony in relation to conversational dynamics.

To conclude, analyzing short excerpts of virtual clinical interviews using automated approaches to extract interpersonal head synchrony revealed that individuals at CHR for psychosis exhibit reduced strength of synchrony in head-nodding movements compared to healthy individuals. This study serves as a proof of concept for the application of automated methods in the analysis of virtual social interactions and provides new insights into alterations in social functioning in this population. Our results indicated that a stronger interpersonal head synchrony was associated with greater social and role functioning. These findings further support the link between motor and social domains and hold promise not only for research but also for developing screening tools, psychosis risk-detection methods, and virtual assessments of social functioning. Longitudinal studies are now needed to track interpersonal head synchrony across different stages of illness, which could help determine whether these changes correspond with symptom progressions or treatment response.

## Supporting information

10.1017/S0033291725102754.sm001 Lozano-Goupil et al. supplementary material Lozano-Goupil et al. supplementary material

## Supplementary material

The supplementary material for this article can be found at http://doi.org/10.1017/S0033291725102754.
