# Supplementary material for: Reduced interpersonal head synchrony in youth at clinical high risk for psychosis
Source: Psychol Med. 2025 Dec 29;55:e387. doi: 10.1017/S0033291725102754 (PMC13058645; doi:10.1017/S0033291725102754)
Supplement: Lozano-Goupil et al. supplementary material [file S0033291725102754sup001.docx]

**Supplementary Materials**

Table S1 - Detailed number of clinical interviews per Assessor and Group

Figure S1 - Boxplot of the strength of interpersonal head synchrony between groups and assessors.

Table S2 **-** Linear mixed models for the strength and delay of synchrony estimate for a **5 sec** window size.

Table S3 - Linear mixed models for the strength and delay of synchrony estimate for a **7 sec** window size.

**Table S1 –** *Detailed number of clinical interviews per Assessor and Group*

| **Assessor** | **Group** | | | **Total** |
| --- | --- | --- | --- | --- |
|  | **HC** | **SUB** | **CHR** |  |
| **1** | 1 | 3 | 2 | 6 |
| **2** | 1 | 8 | 3 | 12 |
| **3** | 2 | 0 | 1 | 3 |
| **4** | 1 | 0 | 0 | 1 |
| **5** | 1 | 0 | 0 | 1 |
| **6** | 1 | 0 | 0 | 1 |
| **7** | 1 | 0 | 0 | 1 |
| **8** | 0 | 1 | 0 | 1 |
| **9** | 1 | 0 | 0 | 1 |
| **10** | 0 | 1 | 0 | 1 |
| **11** | 0 | 2 | 0 | 2 |
| **12** | 2 | 0 | 2 | 4 |
| **13** | 1 | 0 | 1 | 2 |
| **14** | 0 | 0 | 2 | 2 |
| **15** | 0 | 1 | 0 | 1 |
| **16** | 1 | 0 | 2 | 3 |
| **17** | 0 | 0 | 1 | 1 |
| **18** | 0 | 1 | 1 | 2 |
| **19** | 1 | 1 | 0 | 2 |
| **20** | 0 | 0 | 2 | 2 |
| **21** | 1 | 3 | 6 | 10 |
| **22** | 3 | 3 | 1 | 7 |
| **23** | 4 | 4 | 5 | 13 |
| **24** | 0 | 1 | 0 | 1 |
| **25** | 2 | 1 | 5 | 8 |
| **26** | 0 | 0 | 3 | 3 |
| **27** | 1 | 0 | 3 | 4 |
| **28** | 2 | 2 | 3 | 7 |
| **29** | 1 | 1 | 0 | 2 |
| **30** | 1 | 1 | 2 | 4 |
| **31** | 1 | 0 | 2 | 3 |
| **32** | 0 | 2 | 3 | 5 |


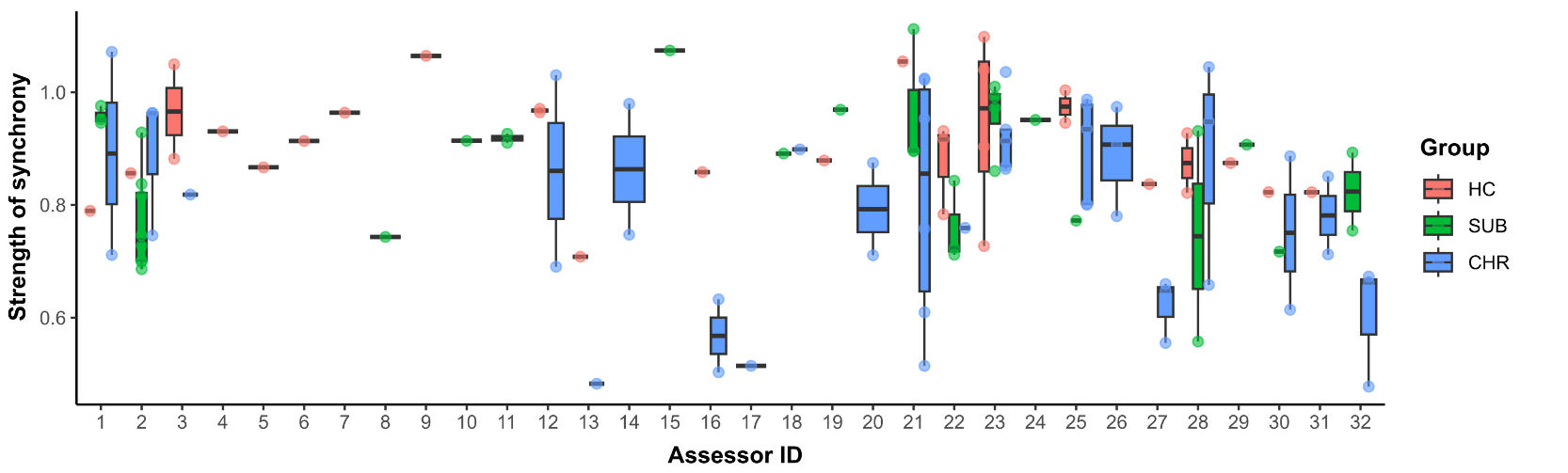


**Figure S1** – Boxplot of the strength of interpersonal head synchrony between groups and assessors.

**Table S2 –** Linear mixed models for the strength and delay of synchrony estimate for a **5 sec** window size.

|  | **Fixed effects** | | | **Random effects** | | |  |
| --- | --- | --- | --- | --- | --- | --- | --- |
| **Strength of synchrony** | Estimates | 95% CI | p | Variance | SD | ICC |  |
| Intercept | 0.85 | [0.81 – 0.88] | **< .001** |  |  |  |  |
| Group [SUB] | -0.02 | [-0.06 – 0.02] | .252 |  |  |  |  |
| Group [CHR] | -0.05 | [-0.08 – -0.02] | **.004** |  |  |  |  |
| Participant Sex [M] | -0.01 | [-0.05 – 0.02] | .464 |  |  |  |  |
| Assessor Sex [M] | 0.03 | [-0.01 – 0.08] | .095 |  |  |  |  |
| Participant Sex [M]x Assessor Sex [M] | -0.03 | [-0.09 – 0.03] | .283 |  |  |  |  |
| Assessor |  |  |  | 0.0005 | 0.023 | 0.096 |  |
| Residual |  |  |  | 0.0050 | 0.071 |  |  |
| **Delay of synchrony** |  | | |  | | |  |
| Intercept | | 0.04 | [-0.9 – 0.17] | .546 |  |  |  |
| Group [SUB] | | -0.07 | [-0.22 – 0.08] | .366 |  |  |  |
| Group [CHR] | | -0.03 | [-0.17 – 0.11] | .698 |  |  |  |
| Participant Sex [M] | | 0.04 | [-0.19 – 0.11] | .609 |  |  |  |
| Assessor Sex [M] | | -0.09 | [-0.24 – 0.06] | .258 |  |  |  |
| Participant Sex [M]x Assessor Sex [M] | | 0.00 | [-0.23 – 0.24] | .985 |  |  |  |
| Assessor | |  |  |  | 0.000 | 0.000 | 0.000 |
| Residual | |  |  |  | 0.093 | 0.305 |  |

**Table S3 –** Linear mixed models for the strength and delay of synchrony estimate for a **7 sec** window size.

|  | **Fixed effects** | | | **Random effects** | | |  |
| --- | --- | --- | --- | --- | --- | --- | --- |
| **Strength of synchrony** | Estimates | 95% CI | p | Variance | SD | ICC |  |
| Intercept | 0.62 | [0.59 – 0.65] | **< .001** |  |  |  |  |
| Group [SUB] | -0.03 | [-0.06 – 0.00] | .092 |  |  |  |  |
| Group [CHR] | -0.04 | [-0.07 – -0.01] | **.005** |  |  |  |  |
| Participant Sex [M] | 0.00 | [-0.03 – 0.04] | .764 |  |  |  |  |
| Assessor Sex [M] | 0.04 | [-0.00 – 0.07] | .070 |  |  |  |  |
| Participant Sex [M]x Assessor Sex [M] | -0.06 | [-0.11 – -0.01] | .**019*** |  |  |  |  |
| Assessor |  |  |  | 0.0006 | 0.025 | 0.142 |  |
| Residual |  |  |  | 0.0039 | 0.062 |  |  |
| **Delay of synchrony** |  | | |  | | |  |
| Intercept | | 0.01 | [-0.16 – 0.18] | .895 |  |  |  |
| Group [SUB] | | -0.12 | [-0.31 – 0.06] | .193 |  |  |  |
| Group [CHR] | | -0.10 | [-0.27 – 0.07] | .267 |  |  |  |
| Participant Sex [M] | | 0.04 | [-0.14 – 0.22] | .646 |  |  |  |
| Assessor Sex [M] | | 0.00 | [-0.20 – 0.20] | .997 |  |  |  |
| Participant Sex [M]x Assessor Sex [M] | | -0.06 | [-0.34 – 0.23] | .688 |  |  |  |
| Assessor | |  |  |  | 0.0109 | 0.104 | 0.076 |
| Residual | |  |  |  | 0.1326 | 0.364 |  |

*Post-hoc analysis revealed that *female participant–male assessor* dyads exhibited significantly higher strength of synchrony compared to *male participant–male assessor* dyads (E = 0.0546, p = .032). This effect was observed only with the 7-second time window and disappeared when using the 5-second or the initial 3.6-second window.
